# 1096. Evaluation of Vancomycin Pharmacokinetics in Intravenous Drug Users

**DOI:** 10.1093/ofid/ofab466.1290

**Published:** 2021-12-04

**Authors:** Sayo Weihs, Gadison Quick, Ivana Bogdanich

**Affiliations:** 1 University Health Truman Medical Centers, Kansas City, Missouri; 2 Truman Medical Centers, Kansas City, Missouri

## Abstract

**Background:**

People who inject illicit drugs (PWID) are 16 times more likely to develop methicillin-resistant Staphylococcus aureus (MRSA) infections including severe infections like bacteremia and endocarditis. Vancomycin is recommended as the drug of choice for empiric and targeted coverage in both severe and non-severe MRSA infections. Pharmacokinetic literature has suggested up to 31% higher renal clearance in intravenous drug users (IVDU) compared to non-IVDUs. This increased clearance may theoretically lead to more frequent sub-therapeutic troughs in otherwise standard dosing schemes. There is a paucity of data examining vancomycin pharmacokinetics following typical dosing schemes in IVDU population.

**Methods:**

This was a single-center, retrospective chart review that examined therapeutic drug monitoring in patients treated with vancomycin between January 1^st^, 2015 through July 31^st^, 2020. Patients were identified as either IVDU or non-IVUD groups based on ICD-9/10 codes. The primary outcome was the difference between mean first vancomycin steady state troughs. Secondary outcomes were differences in time to first therapeutic trough, mean number of days on vancomycin based on infection, rate of acute kidney injury (AKI) after vancomycin, and rate of vancomycin failure.

**Results:**

A total of 158 patients were included in the analysis (77 IVDU vs. 81 non-IVDU). Mean first vancomycin steady state trough were significantly less in IVDU group compared to non-IVDU group (11.85 vs. 13.98 mcg/mL *P* = 0.007). Mean time to first therapeutic trough and mean number of days treated were significantly higher in IVDU versus non-IVDR samples (65.9 vs. 50.2 hours *P* = 0.044 and 5.4 vs. 12.3 days *P* = 0.017, respectively). There was no detectable difference in rates of AKI and vancomycin failure.

Primary outcome graph for patients with IV drug use

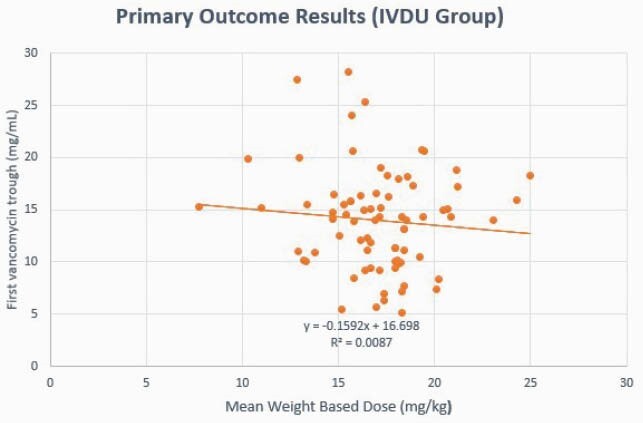

Primary outcome graph for patients without IV drug use

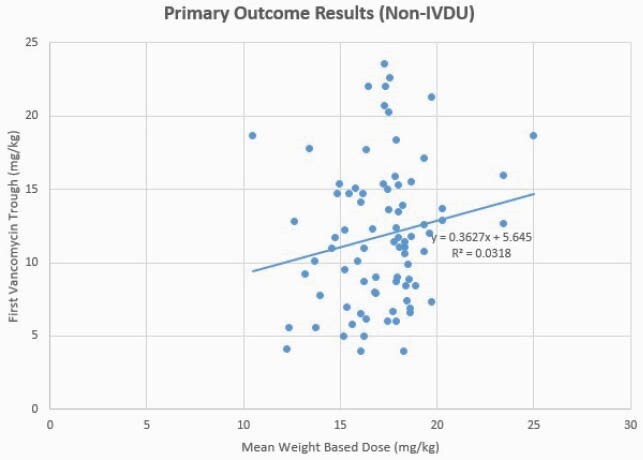

**Conclusion:**

Vancomycin use in patients with IVDU resulted in significantly lower steady state troughs compared to patients who were non-IVDU. These patients also had a longer time to first therapeutic trough. Patient populations who are IVDU may require additional consideration as a special population for future development of vancomycin pharmacokinetic models.

**Disclosures:**

**All Authors**: No reported disclosures

